# Supplementary material for: Immunometabolic Gatekeeping: Reconciling Peto’s & the T-cell Infiltration Prognostic Paradox
Source: ArXiv. 2025 Nov 25:arXiv:2511.20883v1. Preprint. [Version 1] (PMC12676375)
Supplement: 1 [file NIHPP2511.20883V1-supplement-1.pdf]

Supporting materials IA – Single cell gene expression data across normal human tissue (CELLxGENE)

Human single cell gene expression data were downloaded as a .csv file from the CELLxGENE portal (October 2025). The dataset includes expression metrics for various genes across multiple tissues and cell types, including:

i. Tissue and cell type annotations
ii. Gene symbol
iii. Log-normalised mean expression (Expression)
iv. Scaled expression z-scores (Expression, Scaled)
v. Cell count per cell type and tissue
vi. Number of cells expressing a given gene within the respective cell type
vii. Publication/source
viii. Sex

The dataset was read and processed in R (v 4.5.1). The only cancer type listed in **Table 1** for which we did not find a straightforward healthy (i.e., 'normal') tissue baseline was head and neck cancer. While CELLxGENE does have single cell expression data for tissues such as 'Nose' and 'Tongue', calculating composite expression profiles from these tissues was considered methodologically unrepresentative, and thus not included in this analysis.

To examine baseline metabolic versatility in healthy tissue, we focused on fibroblasts and endothelial cells; stromal cell types universally present across tissues and important non-immune cell types in tumour TMEs[156]. As expanded upon in the main text, fibroblasts are often reprogrammed into cancer-associated fibroblasts (CAFs), which actively contribute to the metabolic rewiring and acidification of the tumour microenvironment. Understanding baseline fibroblast and endothelial cell metabolic potential gives insight into how readily a tissue could form an immunosuppressive stromal niche before and/or after transformation.

For any unique combination of Tissue, Cell Type and Gene, the `Number of Cells Expressing Genes` had to be ≥ 10 to meet the QC threshold. Across Cell Type == "fibroblast" | "endothelial cell", gene expression was weighted by the cell type's cell count within each tissue, yielding an average expression per gene that reflects the abundance of each intrinsic cell type in the tissue. All 'focus' tissues (i.e., solid tissues listed in **Table 1**, in accordance with [10]) were represented by ≥5 publications, except for the bladder. Weighted expressions were therefore calculated across 'bladder organ' (2 publications) and 'urinary bladder' (1 publication), to arrive at a total of 3 publications representing single cell expression profiles of normal 'bladder'.

Similarly, T cell exhaustion was gauged by "TOX" expression for Cell Type == "T cell", and subjected to similar data processing as conducted for gauging relative SLC2A1 expression across stromal cells as outlined above (without the need to weight expression across multiple cell types as for this gene we only investigated one cell type).

Moreover, to gauge pH/chemical homeostasis, the expression of a panel of genes was compared: "SLC9A1", "SLC4A2", "SLC4A3", "CA2", "CA9", "CA4", "SLC4A7", "SLC4A4", and "SLC4A5". Weighted expressions were normalised per gene to its cross-tissue maximum to allow balanced cross-tissue comparisons within each marker panel. The normalised values were then averaged across the panel to generate a composite pH-homeostasis score for each tissue. Relative stromal SLC2A1 expression, composite pH-homeostasis score, and T-cell TOX expression were then compared across tissues listed in **Table 1** and categorised into the relative bins shown.

Supporting materials IB – Cross-tissue mitochondrial respiration in normal mice

We used the data supporting figure 1 (elife-96926-fig1-data2-v1) from [12] to create metabolic intensity classifications reported under the column 'Mitochondrial resp. average (complex IV) in mice' in **Table 1.** Complex IV (cytochrome c oxidase) was used as a proxy for mitochondrial respiratory capacity because it is the terminal oxidase of the electron transport chain.
Tissue labels were collapsed into predefined focus categories (tissues analogous to those listed in **Table 1**) as follows: Kidney (Kid-Cortex and Kid-Med), Brain (Cortex), Eye (Eye), Stomach (Stomach), Pancreas (Pancreas), Lung (Lung), Liver (Liver), Fallopian tube (records with tissue "Test Fallop tube" and sex = Female), Skin (Skin), and Colon (Proximal colon and Distal Colon).

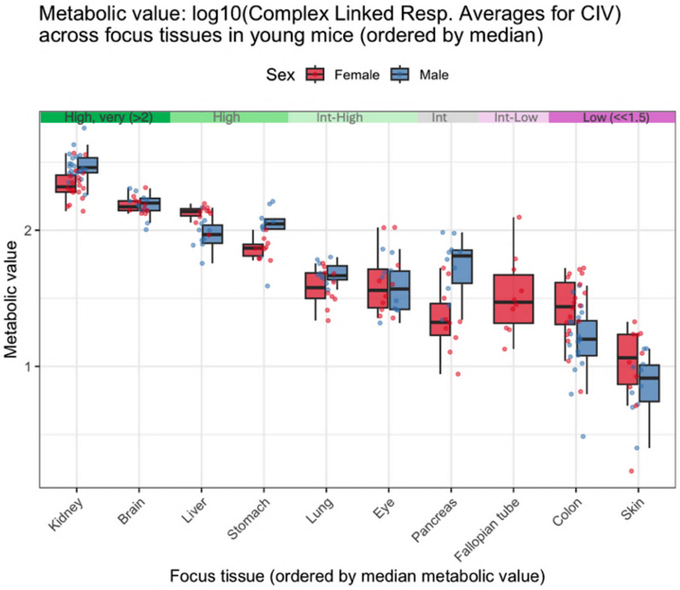

Suppl. Fig. 1. Ranking of tissues (as found in Table 1) by their complex-linked respiratory averages, CIV. Boxplots were ordered by the overall (cross-sex) median metabolic value (log10(Complex Linked Resp. Averages for CIV)).

When using the "Complex Linked Resp. Average" value following normalisation against the tissue's mitochondrial content, "Complex Linked Resp. Avg/MTDR", across all complexes measured (CI, CII, CIV), the ranking remained robust (except for Pancreas having a median lower than that of Fallopian tube and Skin):

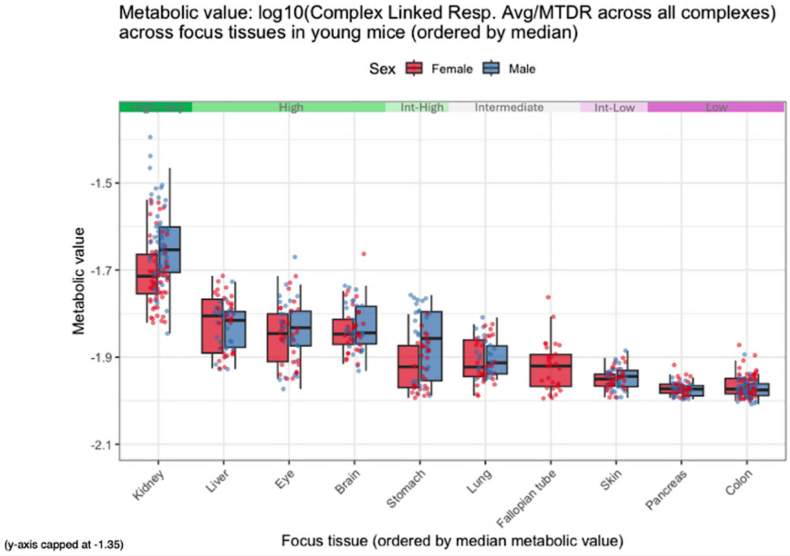

Suppl. Fig. 2. Ranking of tissues by their complex-linked respiratory averages normalised by mitochondrial density (CI, CII, CIV).

Supporting materials II – A minimal diffusion-reaction model illustrating a geometry-driven immunometabolic tipping point at ~3 cm in ccRCC

Overview and clinical motivation
Small (<3 cm) clear cell renal cell carcinomas (ccRCCs) infrequently metastasise, whereas larger tumours show a marked increase in metastatic potential – a phenomenon referred to as the "3 cm rule" [116]. Importantly, this transition typically occurs without acquisition of new driver mutations, suggesting that the shift in metastatic competence reflects microenvironmental changes rather than genetic evolution.

To investigate whether biophysical constraints imposed by tumour geometry may generate such a transition, we constructed a steady-state diffusion-reaction model describing lactate accumulation, oxygen availability, and cytotoxic immune cell infiltration within spherical tumours of varying radii.

Biological rationale
Constitutive HIF stabilisation following VHL pathway loss renders ccRCCs highly glycolytic even under normoxia, leading to continuous production of lactate and protons. Because metabolite production scales with tumour volume, whereas clearance occurs across the tumour surface, larger tumours accumulate metabolic waste more rapidly. This geometric imbalance reduces the surface-area-to-volume (SA:Vol) ratio ($3/R$), limiting oxygen delivery and immune access while impairing clearance of lactate.

We therefore hypothesised that as tumours grow, they cross an immunometabolic tipping point: a transition from an immune-permissive state to an immune-exhausted state driven by altered diffusion geometry. The goal of this model is not to capture all microenvironmental complexity, but to test whether geometry alone can recapitulate the observed size-dependent shift in immune dysfunction and metastatic propensity.

Model set-up
For simplicity, the tumour is assumed to be a perfect sphere of radius R. We solve the model in 1D radial space. For each tumour radius R, the volume $Vol = \frac{4}{3}\pi R^3$ and surface area $SA = 4\pi R^2$ were used to calculate the surface area to volume (SA:Vol) ratio ($3/R$), which modulates boundary condition scaling (e.g., oxygen and cytotoxic immune availability, lactate clearance).

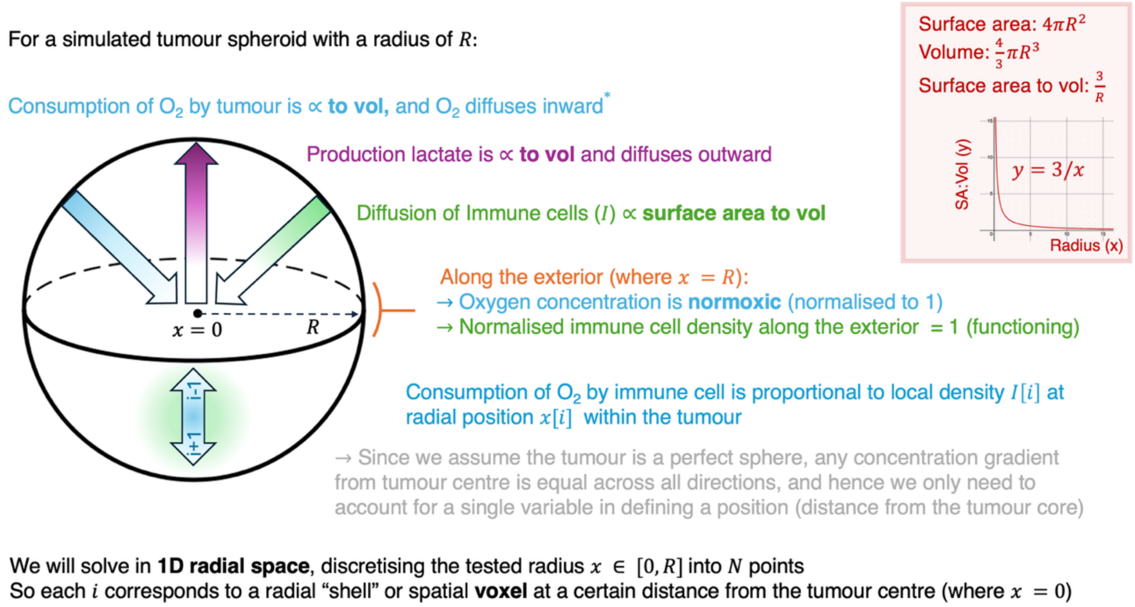

Suppl. Fig. 3. Conceptual overview of model used. Cytotoxic immune infiltration was assessed along a tumour spheroid for different tested radii, ranging from 0.5 cm to 5 cm. Since all tumour cells were assumed to be metabolically active, the tumour's total oxygen consumption was made proportional to the volume, while oxygen supply was assumed to diffuse inward, via the tumour surface. We assumed the

tumour boundary is well-oxygenated/normoxic, as a proxy for exchange with surrounding tissue vasculature*. Production of lactate was assumed to be proportional to tumour volume as well, while diffusion of functional/cytotoxic immune cells was assumed to be proportional to the surface area to volume ratio (SA:Vol). Immune cells were assumed to consume oxygen locally.

*Unlike intratumoural vessels, the normal tissue surrounding the tumour retains functional perfusion, and thus provides a reliable source of oxygen diffusion at the tumour boundary. Tumours often coopt neighbouring vessels, further justifying oxygen diffusion from surrounding tissue into the tumour.

The radial domain was defined as $x \in [0, R]$, where R is the tested tumour radius (tested from 0.5 – 5 cm). The domain was discretised into $N = 400$ evenly spaced points (Δx), and all spatial derivatives were approximated using central finite differences. For a radial coordinate $r = x[i]$, with step size dx, the following steady state equations were solved:

The following steady-state equations were solved iteratively for each metabolic 'species':

1) Lactate ($L(r)$, uniformly produced throughout tumour, diffusing outward);

$$\frac{1}{r^2}\frac{d}{dr}\left(r^2\frac{dL}{dr}\right) + \frac{p_L}{D_L} = 0 \qquad (S1)$$

Where p_L is the lactate production rate, which is scaled with tumour volume, and D_L is the lactate diffusion constant.

2) Immune cells ($I(r)$, diffuse inwards from tumour boundary, suppressed by lactate and/or hypoxia);

$$\frac{1}{r^2}\frac{d}{dr}\left(r^2\frac{dI}{dr}\right) - \frac{1}{D_I}\left(a_L L + a_O H(O)\right)I = 0 \qquad (S2)$$

Where D_I is the immune cell diffusivity (inferred to be low due to size and e.g., stromal barriers), a_L is the suppression due to lactate (/acidosis of co-secreted H^+), a_O is the suppression due to true hypoxia, and $H(O)$ is the hypoxia indicator function, which is 1 if $O < O_{thresh}$ (hypoxia threshold), and 0 otherwise. O_{thresh} was drawn from a uniform distribution $U(0.1,0.5)$, representing 10-50% of normoxic oxygen concentrations. Immune density $I(x)$ is a dimensionless variable representing the relative fraction of functional/cytotoxic immune density, where 1 represents healthy/active state, and 0 represents complete suppression/inactivation.

3) Oxygen ($O(r)$, diffuses from boundary inward, consumed by tumour and immune cells)

$$\frac{1}{r^2}\frac{d}{dr}\left(r^2\frac{dO}{dr}\right) - \frac{1}{D_O}\left(p_T + p_I I\right) = 0 \qquad (S3)$$

Where D_O represents oxygen's diffusion (which is assumed to be faster than diffusion of the others), p_T is oxygen consumption by tumour cells, which is scaled with tumour volume, while p_I is local consumption of oxygen by immune cells (not scaled with volume but dependent on local I)

These PDEs were discretised using central finite differences on a radial mesh:

$$L_i = \frac{\left(r_{i+\frac{1}{2}}\right)^2 L_{i+1} + \left(r_{i-\frac{1}{2}}\right)^2 L_{i-1} + \frac{\Delta x^2 p_L r_i^2}{D_L}}{\left(r_{i+\frac{1}{2}}\right)^2 + \left(r_{i-\frac{1}{2}}\right)^2} \qquad (S4)$$

$$I_i = \frac{\left(r_{i+\frac{1}{2}}\right)^2 I_{i+1} + \left(r_{i-\frac{1}{2}}\right)^2 I_{i-1}}{\left(r_{i+\frac{1}{2}}\right)^2 + \left(r_{i-\frac{1}{2}}\right)^2 + \frac{\Delta x^2 r_i^2}{D_I}\left(a_L L_i + a_O H_i\right)} \qquad (S5)$$

$$O_i = \frac{\left(r_{i+\frac{1}{2}}\right)^2 O_{i+1} + \left(r_{i-\frac{1}{2}}\right)^2 O_{i-1} - \frac{\Delta x^2 r_i^2 (p_T + p_I I_i)}{D_O}}{\left(r_{i+\frac{1}{2}}\right)^2 + \left(r_{i-\frac{1}{2}}\right)^2} \qquad (S6)$$

Parameters were either fixed or randomly sampled:

1) Diffusion coefficients were set apart by orders of magnitude to represent relatively faster diffusion for smaller 'species', such that $D_O = 10, D_L = 1, D_I = 0.01;$
2) Immune cell suppression parameters were drawn from uniform distributions: $a_L | a_O \sim U(0.1, 0.9)$
3) Production rate of lactate and consumption rate of oxygen by the tumour scaled with volume, such that $p_L = p_{L0} Vol,\ p_T = p_{T0} Vol$

Boundary & initial conditions

Boundary conditions were informed by geometric constraints, such that:

1) Oxygen and cytotoxic immune cell access at the tumour boundary were scaled by the SA:Vol ratio, under the rationale that as tumours grow, a relatively smaller surface is 'responsible' for supplying oxygen and immune access to a growing mass;

$$O_{ext} = O_{base}\frac{SA}{Vol};\quad I_{ext} = I_{base}\frac{SA}{Vol} \quad (S7)$$

We set both O_{base} and I_{base} to 1, representing normoxia and healthy/functional immune density, respectively.

2) Lactate clearance at the tumour boundary (i.e., peritumoural region) was scaled inversely with the SA:Vol ratio, to simulate impaired metabolite clearance in larger tumours;

$$L_{ext} = \max\left(0, 1 - \frac{SA}{Vol}\right) \quad (S8)$$

This resulted in greater lactate accumulation in larger tumours, mimicking the biophysical constraint in diffusion and thus impaired lactate clearance in larger tumours.

Boundary conditions were implemented using ghost points with mirror reflection for the core (i.e., $L[1] = L[2]$, etc.).

For each tumour radius tested, the system was initialised with $L = 0, I = 0.1, O = 1$. Thus, the model's starting condition reflects no pre-existing lactate buildup by the tumour, low-level basal immune infiltration throughout the tumour upon initiation, and complete oxygenation throughout the tumour, respectively. Biologically, this models cytotoxic immune cells as already present or attempting to access the tumour, even if diffusion and suppression have not yet taken full effect. Iterative updates were then applied to solve the system until convergence.

Numerical solving

The steady-state equations were solved using a fixed-point iterative scheme on the discretised radial grid, updating each species until convergence (tolerance $<10^{-4}$). This approach is equivalent to integrating the diffusion-reaction equations forward in time to steady state but avoids explicit time stepping. All simulations were implemented in R (v 4.2.3).

Each tumour radius was simulated over 50 independent replicates with resampled parameters to account for parameter stochasticity (of those drawn from uniform distributions).

For comparison, we simulated control conditions where we assumed no hypoxia-induced or lactate-induced limitation on immune cell activity/migration/recruitment via $a_O = 0$ or $a_L = 0$, respectively.

Outcome metric

Then, for each round of integrations, we calculated a Immune Dysfunction Score (IDS), which was defined as the fraction of tumour volume where functional/cytotoxic immune cell density $I(x)$ fell below a critical threshold of I_{thresh}, which we set to 0.2, representing immune exhaustion/suppression (<20% functional cytotoxic cell density). This metric was computed across a range of tumour radii $R \in [0.5, 5.0]$ cm in 0.25 cm increments. The IDS fraction was summarised (median) across replicates.

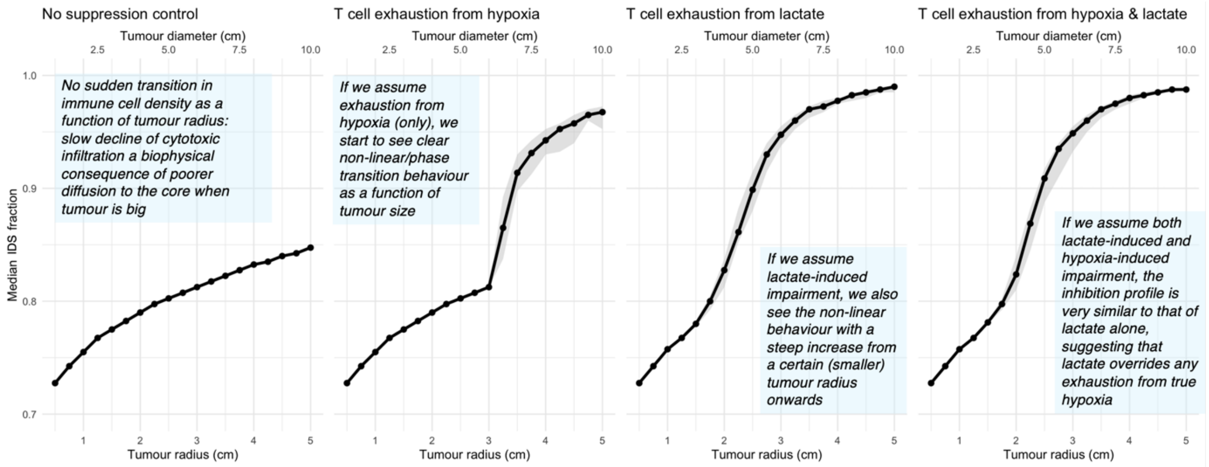

Suppl. Fig. 4. Model outcomes for simulations with and without hypoxia-induced and/or lactate-induced immune cell exhaustion.
Note that for the hypoxia penalty, due to the nature of the indicator function, the transition is more sudden compared to how the model accounts for lactate impairment (which was proportional to lactate concentration, whereas hypoxia-related T cell exhaustion occurs when oxygen drops below the hypoxia boundary).

Results & Discussion

The model predicts that as tumours enlarge, diffusion geometry alone can trigger a sharp transition from active immune function to immune exhaustion, driven by accumulation or impaired clearance of glycolytic byproducts such as lactate (**Suppl. Fig. 4**). This transition emerges without invoking new genetic alterations, suggesting that the clinical “3 cm rule” reflects a breakdown of immune-metabolic equilibrium rather than a mutational threshold. Within this framework, small tumours remain in a quasi-stable state where immune surveillance and metabolism are balanced, while larger tumours accumulate lactate and acidosis, generating an immunosuppressive niche that enables metastatic competence.

Importantly, the emergence of an exhaustion threshold occurred even when hypoxia was removed from the model, indicating that size-dependent lactate accumulation alone can generate an immune-suppressive niche. However, the model assumes spherical symmetry, uniform metabolic activity, steady-state conditions, and a single effective immune population (i.e., no explicit ‘split’ of immune cell population into immune exhausted population). It does not incorporate explicit angiogenesis, stromal barriers, or T cell-derived lactate, although the latter is qualitatively captured by the lactate-dependent immune suppression term.

Thus, results should be interpreted qualitatively, illustrating a plausible mechanistic basis by which tumour geometry alone can generate a size-dependent immunometabolic shift.
